# The Labour Ministry and Unemployment Insurance

**Published:** 1921-01-08

**Authors:** 


### The Labour Ministry and Unemployment Insurance

THE inquiry by the Ministry of Labour whether the employment of sisters, staff nurses, and probationers employed in a voluntary hospital is such employment as to bring them under the provisions of the Unemployment Insurance Act was held on Wednesday, January 5, at 8 Richmond Terrace, Whitehall. Mr. B. O. Bircham, Legal Adviser to the Ministry, was in the chair, and made it clear at the outset that the inquiry was being held as regards the three classes of employees specifically mentioned, and though he would not confine the speakers to those cases, he desired them to limit their remarks as far as possible to nurses employed in voluntary hospitals. The hospital authorities represented at the inquiry were Mr. G. Q. Roberts, C.B.E. (St. Thomas's Hospital), Mr. E. W. Morris, C.B.E. (London Hospital), and Mr. J. Courtney Buchanan, C.B.E., honorary secretary of the British Hospitals Association, who also represented St. Bartholomew's Hospital in the absence of Mr. Hayes. The nursing profession was represented by Miss Cox-Davies (Royal Free Hospital), Miss Hogg (Guy's Hospital), Miss Rundle (College of Nursing), Mrs. Bedford Fenwick and Miss Macdonald (Royal British Nurses' Association), Miss Pearse (National Union of Trained Nurses), and Mrs. Richmond and Miss Peterkin (honorary secretary and general superintendent of Queen Victoria Jubilee Nurses' Institute). All the speakers were unanimous that nurses, both those employed in hospital, private nursing, and district nursing, desired exclusion from the Act, which was not calculated to benefit them as individuals or as a profession; in fact, that it would lower the status of nursing and therefore make the profession unattractive to the more educated class and to the right type of woman; and that unemployment insurance is unnecessary for nurses, as there is practically no unemployment amongst them. Individual cases of appeals for exemption (from St. Thomas's, St. Bartholomew's, and the London Hospitals) were also gone into.

The Chairman pointed out that nurses whose rates of remuneration and emoluments exceeded in value £250 a year would be excepted under sub-section (*h*) of section 2, and it was admitted by some speakers that the senior sisters in large hospitals would fall under this exception. He went fully into questions concerning the duties, contracts, and employment of the nursing staff, both in hospital and in private practice. The evidence taken will now doubtless be considered by the Minister, and meanwhile no decision was given, but at the conclusion of the hearing it was pointed out that if the Minister should decide that nurses must be provided for under the Act, it is competent for some Association representing the whole of the profession and the employers to formulate its own special scheme of unemployment insurance under Section 18 of the Act. The Ministry's representative asked if the British Hospitals Association, as representing hospitals generally, had considered this point, and Mr. Buchanan replied that as a body it had not done so, as the object was to give effect to the desire of the nurses to be taken out of the Act. Unemployment insurance is not desired by the profession, and this is the point that was generally insisted on by all those present at the inquiry.

